# Analysis of the posterior cingulate cortex with [18F]FDG-PET and Naa/mI in mild cognitive impairment and Alzheimer's disease: Correlations and differences between the two methods

**DOI:** 10.1590/1980-57642015DN94000385

**Published:** 2015

**Authors:** Artur M.N. Coutinho, Fábio H.G. Porto, Poliana F. Zampieri, Maria C. Otaduy, Tíbor R. Perroco, Maira O. Oliveira, Rafael F. Nunes, Toulouse Leusin Pinheiro, Cassio M.C. Bottino, Claudia C. Leite, Carlos A. Buchpiguel

**Affiliations:** 1 Centro de Medicina Nuclear, Instituto e Departamento de Radiologia, HC/FMUSP, LIM 43.; 2 Serviço de Ressonância Magnética, Instituto e Departamento de Radiologia, HC/FMUSP, LIM 44.; 3 Centro de Referência em Distúrbios Cognitivos (CEREDIC) do HC/FMUSP.

**Keywords:** positron-emission tomography, spectrum analysis, magnetic resonance imaging, mild cognitive impairment, Alzheimer's disease, tomografia por emissão de pósitrons, análise espectral, imagem por ressonância magnética, comprometimento cognitivo leve, doença de Alzheimer

## Abstract

Reduction of regional brain glucose metabolism (rBGM) measured by [18F]FDG-PET in the posterior cingulate cortex (PCC) has been associated with a higher conversion rate from mild cognitive impairment (MCI) to Alzheimer's disease (AD). Magnetic Resonance Spectroscopy (MRS) is a potential biomarker that has disclosed Naa/mI reductions within the PCC in both MCI and AD. Studies investigating the relationships between the two modalities are scarce.

**Objective:**

To evaluate differences and possible correlations between the findings of rBGM and NAA/mI in the PCC of individuals with AD, MCI and of cognitively normal volunteers.

**Methods:**

Patients diagnosed with AD (N=32) or MCI (N=27) and cognitively normal older adults (CG, N=28), were submitted to [18F]FDG-PET and MRS to analyze the PCC. The two methods were compared and possible correlations between the modalities were investigated.

**Results:**

The AD group exhibited rBGM reduction in the PCC when compared to the CG but not in the MCI group. MRS revealed lower NAA/mI values in the AD group compared to the CG but not in the MCI group. A positive correlation between rBGM and NAA/mI in the PCC was found. NAA/mI reduction in the PCC differentiated AD patients from control subjects with an area under the ROC curve of 0.70, while [18F]FDG-PET yielded a value of 0.93.

**Conclusion:**

rBGM and Naa/mI in the PCC were positively correlated in patients with MCI and AD. [18F]FDG-PET had greater accuracy than MRS for discriminating AD patients from controls.

## Abstract (pt)

Redução do metabolismo cerebral regional glicolítico (MRG) medido pela PET-18FDG no giro do cíngulo posterior (GCP) está relacionada a maior conversão para doença de Alzheimer (DA) em sujeitos com comprometimento cognitivo leve (CCL). Espectroscopia por ressonância magnética (MRS), um biomarcador promissor, demonstra redução de Naa/mI no GCP na DA. Raros estudos avaliam relações entre Naa/mI e MRG.

**Objetivo:**

Avaliar diferenças e possíveis correlações entre MRG com PET-18FDG e Naa/mI por MRS no GCP de sujeitos com DA, CCL e voluntários normais.

**Métodos:**

Sujeitos com DA (N=32), CCL amnéstico (N=27) e voluntários idosos normais (GC, N=28), foram submetidos a PET-18FDG e análise de Naa/mI no GCP. A performance de ambos os métodos foi então comparada e verificou-se a existência de correlações entre os achados da PET e da MRS.

**Resultados:**

Observou-se hipometabolismo glicolítico nos pacientes com DA no GCP em relação ao GC, porém não no CCL. A MRS demonstrou valores menores de Naa/mI no CP do grupo DA em relação ao GC, porém também sem diferenças entre CCL e GC. A área sob a curva ROC demonstrou valor de 0,70 para MRS e 0,93 para o MRG no GCP para diferenciar DA do GC. Houve correlação positiva entre o MRG e o Naa/mI no GCP.

**Conclusão:**

Os valores de metabolismo de glicose à PET e de Naa/mI à MRS no giro do cíngulo posterior apresentaram correlação positiva estatisticamente significante na presente amostra. Houve ainda superioridade da PET-18FDG para diferenciar DA do GC.

## INTRODUCTION

Alzheimer's disease (AD) has become a public health problem with the rise in life expectancy, since there is currently no treatment that modifies its progression.^1-3^ Correct diagnosis in the early stages of the disease is crucial to better understand its pathophysiology and to develop treatments to slow its progression. Mild cognitive impairment (MCI), especially the amnestic subtype, is a symptomatic transitional state from normal aging to early dementia. MCI is characterized by subjective memory complaints and objective decline in cognitive performance, with normal or near-normal functional activities of daily living.^4,5^

Positron emission tomography using [18F]fluorodeoxyglucose ([18F]FDG-PET) is a well-established tool for monitoring regional brain glucose metabolism (rBGM). A progressive reduction of rBGM in specific areas occurs years before the onset of clinical symptoms in patients with verified AD and during the MCI phase, particularly in the temporoparietal cortex and posterior cingulate cortex (PCC) association.^6-10^ Of all the areas, the PCC seems to be the most sensitive marker for predicting which patients with MCI will progress to AD.^7,10,11^

Magnetic Resonance Spectroscopy (MRS) uses a standard MRI scanner and acquires a spectrum that expresses metabolite concentrations in the brain. It is a potentially useful noninvasive neuroimaging technique for detecting brain biochemical changes associated with neurodegenerative diseases.^12^ MRS has potential utility as a biomarker in MCI and early dementia, helping with early (and differential) diagnosis and tracking disease progression surveillance.^13-15^

Some metabolites commonly studied with MRS and present at high concentrations in the brain are: N-Acetyl Aspartate (NAA), choline (Cho), creatine (Cr), myo-inositol (mI), glutamate and glutamine (Glx).^16^ Each metabolite is sensitive to different processes in the brain. MRS studies have shown decreased NAA/mI and increased mI/Cr ratios in the brain of subjects with MCI, including in the PCC, which may correspond to neuronal injury.^13,14^ NAA is mainly found in neurons, and thus NAA reduction reflects neuronal loss or dysfunction; mI is a marker of glial cells, thus its concentration depends on the quantity of gliosis.^16-18^ One of the drawbacks of the method, however, is the need for manually drawn regions of interest (ROI) in different areas of the brain, since values of Naa/mI may vary among different brain regions, i.e. the PCC and the hippocampus. Values measured can also vary according to operator experience.^16^

Although hypometabolism in the PCC measured by [18F]FDG-PET is a classical biomarker of disease progression to AD in MCI and some MRS results disclose early neuronal injury in this area, studies correlating the findings of the two modalities are scarce. Given these methods theoretically reflect correlated biologic processes, this study sought to investigate whether the two measures are closely related in elderly patients with AD or amnestic MCI and control subjects without cognitive complaints.

Thus, the objectives of this study were to assess possible differences in findings on [18F]FDG-PET and in NAA/mI ratio (a measure of neuronal injury) assessed by MRS in the PCC among patients with AD or MCI and cognitively normal volunteers, and also to determine possible correlations between the two methods in the PCC of these individuals.

## METHODS

**Participants.** Older adults (≥60 years old) with subjective cognitive complaints were recruited from the Cognitive Disorders Reference Center (CEREDIC) of our hospital. Patients had to have reported cognitive complaints, confirmed by a collateral source, usually a relative or spouse. All participants underwent complete neurological and psychiatric evaluation as well as comprehensive neuropsychological tests. The final diagnosis was established by consensus of at least two physicians (neurologists or psychiatrists) with expertise in cognitive and behavioral neurology. The healthy older adults without cognitive complaints were recruited in the community or from a *pool* of cognitive normal older subjects from our Institution to serve as members of the control group. After the initial work-up, participants were classified into one of three groups: Alzheimer's disease group (AD), mild cognitive impairment group (MCI) or control group (CG).

Patients from the AD group were diagnosed according to the DSM-IV and the NINCDS-ADRDA criteria.^19^ The revised Petersen criteria were used to diagnose individuals with MCI.^4,5^ Only patients with amnestic MCI were included. Severity of the cognitive complaints was measured by the Clinical Dementia Rating (CDR) scale.^20^ Only individuals with a score of 1.0 on the Clinical Dementia Rating were included in the AD group (defined as early AD). All subjects from the MCI and Control groups had CDR=0.5 (MCI) and CDR=0 (CG), respectively.

All subjects were submitted to the Mini-Mental State Examination,^21^ the Brief Cognitive Screening Battery (BCSB),^22^ the Dementia Rating Scale^23,24^ and to a comprehensive neuropsychological evaluation, which included the following tests: Visual Reproduction subtest of the Wechsler Memory Scale - Revised (WMS-R),^25^ Rey Complex Figure - delayed recall,^26^ Logical Memory subtest of the Wechsler Memory Scale - Revised (WMS-R),^25^ Selective Reminding Test,^27^ Block Design subtest - Wechsler Adult Intelligence Scale (WAIS),^28^ Rey Complex Figure copy,^26^ attention/executive functions (Trail Making Test A and B),^26^ and phonemic verbal fluency (F.A.S.),^26^ and language (semantic verbal fluency - supermarket).^23,24^ The application, scoring and interpretation of the results obtained for all tests were performed according to their respective reference guides. All brain-imaging procedures were performed within 2 weeks of the clinical examinations and neuropsychological testing.

Exclusion criteria included:

[1] volunteers with clinically relevant psychiatric symptoms meeting DSM-IV criteria;
[2] any uncompensated clinical comorbidity, such as cardiac failure or anemia;
[3] history or presence of signs of other neurologic diseases, such as Parkinson's disease, epilepsy, inflammatory disease or stroke, with the exception of migraine;
[4] presence of any drug abuse (especially alcoholism);
[5] patients with diabetes mellitus without adequate glycemic control in the last two weeks;
[6] demented subjects with CDR >1.0;
[7] presence of neoplastic or significant vascular lesions on the MRI, according to the judgment of an assistant neuroradiologist and of the authors (AMNC);
[8] contraindication of the MRI exam. Antidepressant use was not strictly exclusionary; individuals using antidepressants were allowed to participate if on a stable dose for at least three months and without symptoms of an active psychiatric disease at the time of screening.

This research project was approved by the ethics committee of the Hospital das Clínicas da Faculdade de Medicina da Universidade de São Paulo, and complied with the provisions of the Declaration of Helsinki. All subjects signed a consent form.

**Magnetic resonance imaging acquisition.** All patients underwent a standard brain MRI scan to exclude the presence of significant lesions and for co-registration with [18F]FDG-PET images.

**Figure 1:**
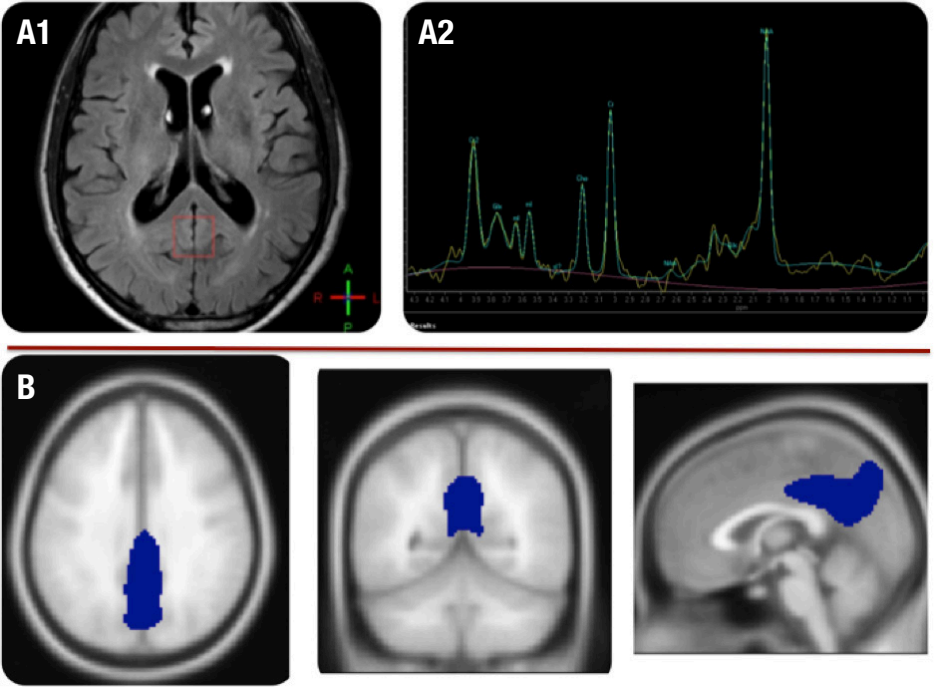
Illustration of the regions of interest on MRS and [18F]FDG-PET. [A1] ROI in posterior cingulate drawn in the FLAIR sequence of MRI (red square); [A2] different peaks calculated on MRS; [B] (lower row): ROI in PCC of [18F]FDG-PET images, drawn with the SPM8 MarsBar toolbox.

Brain MRI exams were performed on a 3.0T magnetic resonance scanner (Intera Achieva, PHILIPS Healthcare, Best, The Netherlands) with an 8-channel head coil and the imaging protocol included the following sequences: 3D-T1 Fast Field Echo (3D-T1 FFE), axial T2-weighted fast spin echo (FSE), axial fluid-attenuated inversion recovery (FLAIR), coronal T2- weighted fast spin echo (FSE) with fat saturation (SPIR), and diffusion. Finally, a single-voxel 1H-MRS was obtained from the PCC using the PRESS sequence with 128 averages, TR of 1500 ms and TE of 35 ms. Voxel size was 2×2×2 cm^3^ and placed in the PCC (Figure 1). NAA and mI concentrations were quantified relative to an internal water reference using LCModel.^29^

**Positron emission tomography imaging acquisition.** Patients with blood glucose levels lower than 180 mg/mL and at least 4 hours of fasting received an intravenous injection of 370 MBq of [18F]FDG in a peripheral vein, and rested with eyes open and ears unplugged for 60 minutes in a calm, silent and slightly darkened room. Images were acquired using a Siemens Biograph PET-CT scanner (CTI/ Siemens, Knoxville, TN, USA).

PET data was analyzed on a voxel-by-voxel basis using the SPM8 software program (Wellcome Department of Cognitive Neurology, Functional Imaging Laboratory, London, UK) in conjunction with MATLAB R2009a (The Mathworks Inc., U.S.A.). Each PET study was co-registered with the individuals' respective MRI images (volumetric T1) and spatially normalized in SPM8 into a standard stereotactic space, based on the SPM8/Montreal Neurologic Institute (MNI) space. Global uptake differences between brain scans were adjusted using the "proportional scaling" SPM option. The relevant peak voxels were identified in terms of coordinates according to Talairach and Tournoux with the aid of the Talairach Client software, and after conversion from the SPM/MNI space. Complete details of the [18F]FDG-PET acquisition and imaging processing have been described previously.^30,31^

**Statistical analysis and [18F]FDG-PET ROI definition.** An analysis of variance (ANOVA) test was used to search for regional brain glucose metabolism (rBGM) differences across the groups (AD, MCI and CG) using the SPM software. Post-hoc analyses with unpaired T-tests were used to examine differences between each pair of groups. SPM8 maps were generated with a visualization threshold of p<0.001and the threshold for significance at the voxel level was set at p=0.001 (Z score=3.09) with a minimum extension of 10 voxels in the corresponding cluster. The initial exploratory analyses with SPM maps generated a t statistic for each voxel, thus constituting statistical parametric maps.

In order to obtain values of the radioactive counts related to the rBGM in the PCC as measured with [18F]FDG-PET, a direct analysis of this region was performed with SPM, adopting the small volume correction approach (SVC). After identifying the cluster with rBGM reduction in the PCC in the AD group, a volumetric region of interest (ROI) of this cluster was generated (Figure 1). In order to increase the specificity of this analysis, the statistical cutoff was set at p<0.05, corrected for multiple comparisons with the familywise error method (p_FWE_), with a minimum extension of 20 voxels in the corresponding cluster. Subsequently, numeric values representing [18F]FDG uptake measures in that cluster for each individual in all groups (after the whole normalization process) were extracted with the toolbox MarsBar for SPM (http://marsbar.sourceforge.net/) under the option "explore design/files and factors".^32^

Demographic data and the values of the MRS NAA/mI ratio in the PCC were compared across groups by an ANOVA analysis with the aid of SPSS software version 17.0 (SPSS Inc., Chicago IL).

After obtaining the average radioactive counts in the PCC and the NAA/mI ratio of all subjects, numeric data were assessed with the SPSS software to identify possible correlations among the data. Sensitivity and specificity curves for each method were also generated in order to compare the diagnostic performance of the two approaches.

## RESULTS

Eighty-seven (87) individuals were included and classified into one of the three groups: AD (n=32), MCI (n=27) and CG (n=28). Demographic data for the sample is shown in Table 1. Subjects included in the CG were younger (p<0.001) than those from the AD group, had more years of education than both patient groups (p=0.001 for MCI and p<0.001for AD) and also higher Mini-Mental State Examination (MMSE) scores than both the MCI (p=0.031) and AD (p<0.001) groups. Performance on the MMSE was also higher in the MCI group than in the AD group (p<0.001).

**Table 1:** Demographic data for the sample.

	CG=28 (CDR=0) Mean (SD)	MCI=27 (CDR=0.5) Mean (SD)	AD=32 (CDR=1.0) Mean (SD)	P (two-tailed) Multiple comparison
	**69.7 (6.6)**	72.7 (6.8)	76.3 (6.7)	**0.001** **CG × AD**
Gender (F/M)**	6 / 22	12 / 15	10 / 22	p>0.05
Education (Y)*	**12.8 (5.1)**	8.0 (4.9)	7.1 (4.1)	**<0.001** CG × AD (<0.001) & CG × MCI (p =0.001)
MMSE*	**29.0 (1.0)**	27.2 (2.1)	22.9 (3.4)	<0.001

CG × AD (<0.001); MCI × AD (p<0.001) & CG × MCI (p =0.031).

* ANOVA (Post-hoc test: Bonferroni);

** Chi-Square; AD: Alzheimer's disease; CG: control group; F: female; M: male; MCI: Mild cognitive impairment; MMSE: Mini-Mental State Examination; SD: Standard deviation; Y: Years.

**Figure 2:**
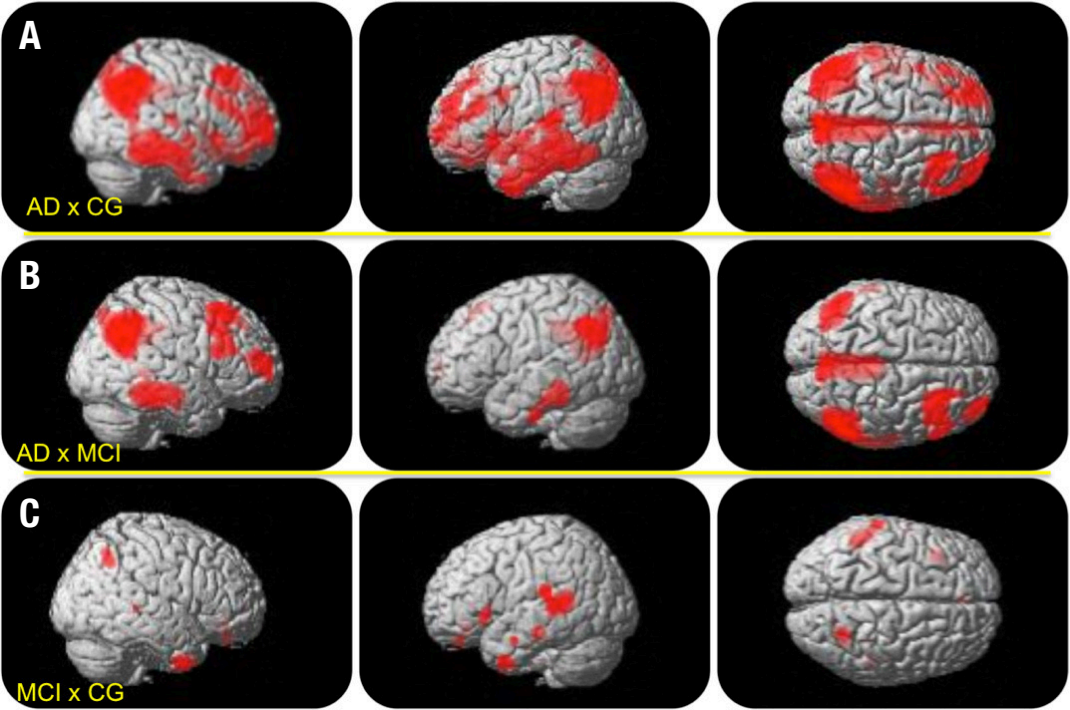
Illustrative anatomic location of peak voxels of rBGM reductions as measured by [18F]FDG-PET. [A] (upper row): areas of rBGM reduction in the AD group versus the CG in large areas of the temporoparietal cortex, posterior cingulate and prefrontal cortex; [B] (middle row): areas of rBGM reduction in the AD group versus the MCI group, showing hypometabolism in similar areas seen in A, albeit with lesser extension and intensity; [C] areas of rBGM reduction in the MCI group versus CG, restricted to the temporal lobes and temporo-parietal association cortex, without significant changes in the PCC.

The AD group exhibited rBGM reduction in large areas of the PCC and temporoparietal cortex compared to the CG, but also in less evident areas of the frontal cortex. This metabolism reduction existed in similar areas among the MCI patients, albeit with lesser extension. The majority of these areas persisted after corrections for multiple comparisons using the FWE method (pFWE<0.001). The MCI individuals showed rBGM reduction in the temporal association cortex in relation to CG (p<0.001)(not surviving correction for multiple comparisons) that was more restricted to the temporal lobes compared to the hypometabolism seen in the AD group. The SVC analysis of the PCC depicted no differences between the MCI group and the CG after correction for multiple comparisons (p_FWE_<0.05). The areas of metabolic reduction are illustrated in Figure 2 (complete statistical results of the SPM8 analysis are beyond the scope of the present work and are not provided).

**Figure 3:**
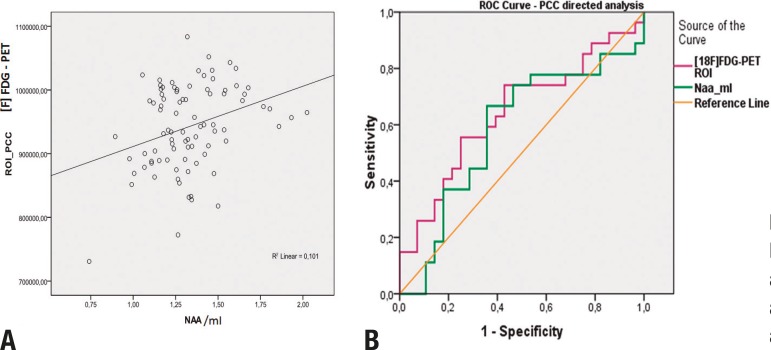
[A] Scatter plot of the correlation curve between values of rBGM (y axis) and Naa/mI (x axis) in the PCC; [B] ROC curves of the directed analysis of the PCC with MRS and Naa/mI peak and with the [18F]FDG-PET ROI analysis.

MRS analysis showed lower NAA/mI values in the AD group compared to the CG (p=0.024). A tendency for lower NAA/mI peak in the PCC was found in the MCI group compared to the CG (p=0.06). This data is also shown in Table 2 and illustrated in Figure 3.

**Table 2:** Summary of key findings of the study.

A – [18F]FDG-PET analysis using SVC of the PCC with SPM8*				
**Comparisons**	**Cluster size (mm^3^)**	**pFWE**	**p**^#^	**Z score**
*AD × CG*				
Right posterior cingulate gyrus	129	**< 0.001**	**< 0.00001**	7.49
Left posterior cingulate gyrus	241	**< 0.001**	**< 0.00001**	7.56
*MCI × CG*	No suprathreshold clusters (p >0.001)			
B – Naa/mI MRS ROI**				
**Comparisons**				**p**
*AD × CG*				**0.024**
*MCI × CG*				0.060
**C – Correlation analysis**				
			**Correlation**	**p**
*[* ^18^ *F] FDG-PET × Naa/mI *			0.361	0.001
**D – ROC curve analysis of the different PCC ROIs**				
			**Area under the ROC curve**	
*[* ^18^ *F] FDG-PET*			**0.935**	
*Naa/mI*			0.708	

* Results at the peak voxel level (ANOVA and post-hoc unpaired t-test);

** ANOVA and post-hoc unpaired t-test with SPSS;

# p value uncorrected for multiple comparisons; pFWE: p value corrected for multiple comparisons with the familywise error method; MCI: Amnestic MCI; CG: Control group; SPM: statistical parametric mapping; SVC: small volume correction method, directed to the PCC.

A positive correlation between rBGM and NAA/mI peak in the PCC was found (r= 0.317; p= 0.012) (Table 2 and Figure 3). Lower NAA/mI in the PCC voxel differentiated AD patients from control subjects, with an area under the receiver operating characteristic curve of 0.70 (CI=0.57-0.84, p =0.006), while the ROI analysis of the PET data yielded a value of 0.93 (CI=0.88-0.99, p<0.001) (Figure 3).

## DISCUSSION

Hypometabolism in the PCC showed good correlation with clinical measures of cognitive impairment such as the CDR sum of boxes.^33^ This reduction is classically related to conversion from MCI to AD and is also considered a standard biomarker for differentiating AD from non-demented subjects.^7,10,34,35^ The areas of rBGM reduction seen in the temporoparietal cortex of AD and MCI groups in comparison to the CG were also previously described as typical areas of neurodegeneration in these conditions.^6,8,9^ The results of the present study confirmed these findings by showing rBGM reduction in the temporoparietal cortex of both AD and MCI groups, albeit with lesser extension and intensity in the latter, as expected. However, the rBGM reduction in the PCC was not statistically significant in the MCI group, thereby failing to corroborate the results of other authors.

The MRS Naa/mI analysis revealed similar results in the AD group, showing a lower Naa/mI ratio for the AD group compared to CG. Naa/mI was also lower in the MCI group, but again did not reach statistical significance compared to the CG. These results failed to corroborate the final results of a related meta-analysis, which found lower values of Naa/mI in MCI subjects.^13^ Some of the articles included in this meta-analysis, however, also found no differences in the Naa/mI ratio in the PCC of MCI subjects, while the lower number of subjects included in the present study should also be taken into account.^13^

On the PCC evaluation, the ROC curve analysis of [18F]FDG was superior than the Naa/mI ratio for discriminating AD subjects from cognitively normal older adults. These results indicate that, although a promising tool for evaluating subjects with cognitive decline, analysis of Naa/mI peak by MRS still lacks the sensitivity of rBGM evaluation with [18F]FDG-PET.

With regard to the MCI group, both methods failed to detect significant differences between the MCI group and the CG in the PCC. [18F]FDG-PET, however, disclosed differences between the MCI and CG groups in other areas. A comprehensive analysis of the whole brain with MRS was not performed since it is technically difficult, representing a limitation of the method.

Which areas first present hypometabolism or atrophy in AD and normal aging remains unclear and a matter of ongoing debate.^36^ While some authors have found hypometabolism in the PCC before other changes in MCI, others have found that blood flow and rBGM reductions in the precuneus and temporoparietal cortex can occur without evident PCC hypometabolism.^31,37^

Some authors also argue that rBGM reduction in MCI could be the indirect result of atrophy and partial volume effect (PVE), especially in the medial temporal lobes,^38^ since atrophy in large areas of the temporal lobes occurs in early AD.^39^ Given our data was not corrected for PVE, this hypothesis could not be tested here and may be considered a limitation of the present study.

Hinrichs et al (2011),^40^ using a machine-learning multi-modal approach, proposed that the combination of different biomarkers is superior to each individually for predicting conversion to AD in MCI. However, [18F]FDG-PET tended to be better than other techniques as a single modality although the authors did not include MRS in their analysis. The present study adds information to the cited study, supporting the notion that [18F]FDG as a single modality is superior to others for detecting neurodegeneration in patients with early AD, especially in the PCC.

Brain glucose metabolism is a surrogate marker of synaptic activity.^41^ Accordingly, metabolism should correlate with measures of neuronal activity and density, such as Naa/mI ratio measured with MRS. This hypothesis was confirmed in the present analysis of the PCC cortex and is the most remarkable finding of the study.

The PCC is a hub of the brain's default mode network and one of the most active parts of the brain in the rest state.^42-44^ According to some theories, this renders the region particularly vulnerable to neuronal injuries and to the deposition of amyloid in the AD neurodegeneration process.^36,42^ Our findings of a positive correlation between rBGM and Naa/mI in the PCC of subjects exhibiting different stages of cognitive function are in line with this hypothesis. This indicates that the hypometabolism seen in AD and MCI in the PCC is proportionally accompanied by a reduction in neuronal density as measured by MRS, which likely indicates neuronal injury.

The present study has some limitations. First, patients from the AD group were older than subjects from the CG a factor that may have had some influence on the results. However, age is a major risk factor for AD and age differences are therefore expected.^1,2^ Also, the present degree of rBGM reduction in the PCC and temporoparietal association cortex is not expected in normal aging.^36^ Thus, it is unlikely that the higher age of the subjects included in the AD group influenced the results of the imaging analysis.

Second, the CG had more years of education than the other groups. Bearing in mind the cognitive reserve hypothesis, education is probably a protective factor for the development of AD and may influence the results of neuropsychological and neuroimaging tests.^36,45^ However, according to this theory, subjects with more years of education would have preserved cognitive performance even if presenting some degree of neurodegeneration.^46-48^ Hence, the subjects in the CG should have lower levels of rBGM in certain areas, with cognitive functioning close to or within the normal range. This was not seen in the present cohort, where the MCI and AD groups presented with significant areas of hypometabolism. Therefore, it cannot be excluded that this factor could have contributed to the lack of differences in the PCC between the CG and the MCI group seen in both methods. Some of the patients with a mild degree of neurodegeneration and higher educational levels could be classified as normal on the clinical tests, yet harbor some degree of degeneration in the PCC. However, this is one the drawbacks of the clinical diagnosis based on neuropsychological testing. This possibility can only be tested by comparing these values in a cohort of MCI and cognitively normal elderly subjects paired by age and years of education or after prospective evaluation of the patients.

In summary, rBGM and NAA/mI ratio in the PCC showed a positive correlation in elderly individuals with AD, MCI and no cognitive impairment. Thus, hypometabolism and neuronal injury are probably directly related in the different phases of the AD pathologic and normal aging process. Both methods proved able to distinguish AD patients from controls when evaluating the PCC, with [18F]FDG-PET providing greater accuracy than Naa/mI.
